# Electricity as a Therapeutic Agent

**Published:** 1873-04

**Authors:** G. W. Frion

**Affiliations:** Surgeon South Brooklyn Dispensary


ART. III.—*Electricity as a Therapeutic Agent.* By G. W. FRION, M. D., Surgeon South Brooklyn Dispensary.

What is Electricity? The name is derived from the Greek and refers to its bright shining power. It was first noticed among the ancients in the substance called amber. It is not a fluid and certainly not matter of any kind. Science has decided the question and revealed that electricity is a state into which any matter may be put; but has yet to define the exact nature of that state.

Sir Isaac Newton, in his optics says, "Bodies act one upon another by the attraction of gravity, magnetism and electricity; and it is not improbable that there may be more attractive powers." It is quite evident from this that he had nothing to offer concerning this agency, but vague conjecture.

The speed of electricity is almost incalculable, and no known velocity on this planet can be compared to it, being 200,000 miles per second, and would take less than a second to pass twice around the world.

As late as 1790 electricity was scarcely known, except among a few savants and men of fashion, such as the Abbie Nablet, Dufay, and others. They taught the doctrine that all objects including the human body, could give out sparks of fire, and compared it to lightning. About the same time an Italian professor of anatomy, Galvani, while experimenting with frogs, taught that the muscles and nerves of animals were reservoirs of the electrical fluid, but only accounted for nerves and muscles, and believed the metallic circuit as only accessory. He knew nothing of what is known as the return shock; and his defects have been realized, and proven of great use to scientific posterity. In one of his experiments, he suspended a skinned frog from his iron balcony by a copper wire, and was very much astonished to see the muscles of the dead body contract violently; the sky at the time being very clear. Alexandra Volta was cotemporary with Galvani, and had many long and exciting controversies, and was the discoverer of the Voltaic Pile in 1800. His theory of electricity was attributed to the contact of two different metals conjoined with animal tissues. 1831–2 Michael Faraday made known to the world his discovery of inductive electricity, which changed the whole course of electrical therapeutics. And on this new theory electric machines were constructed, that were more convenient and reliable than the old Voltaic pile. There are many varieties of batteries now in use in this country, and almost every instrument-maker has adopted some peculiar plan. In the city of New York, The Galvano-Faradic Co., and Dr. Jerome Kidder, stand pre-eminent for their excellent machines. Dr. Kidder uses Smee's elements in conjunction with his apparatus. Whatever be the battery employed, it is necessary to combine several elements, say the zinc pole of the first in connection with the copper, and so on to the last element. Smee's element is economical, convenient and very clean; consisting of a plate of platinum between two plates of zinc which must be immersed in a solution of sulphuric acid and water.

The quantity of electricity generated by any machine is proportioned to the amount of chemical action taking place, as intensity or tension, and is that power that enables it to overcome resistance that may impede the progress of the current, and is now divided into three divisions, viz: Franklinic Galvanic and Faradaic electricity. It is quite common to hear of Faradisation continued current, etc., and there are many persons who have but an imperfect idea of these terms. Fifty years ago it was the fashion to use a machine, either a plate or cylinder of glass, which by rapid friction, produced a peculiar electrical disturbance, the result of which you collect on an insulated brass conductor, and is known as static electricity or Franklinic, named I suppose in honor of our great Franklin, who made many discoveries in this peculiar agency.

There are many modifications and improvements now used, and I have now in use one manufactured by Breton & Brothers, of Paris. Franklinic electricty when used in the form of spark, will produce a stinging sensation, and can be made quite powerful. The galvanic or continuous currents are of two sensations, the lesser current is thrilling and a penetrating sensation, while the other may be increased in intensity in proportion to the chemical force employed, and at the positive pole a copper wire may be kept for any length of time at white heat. A distinguished practitioner of Brooklyn, Dr. Byrne, has invented a most powerful electric cautery battery, which is well adapted to uterine surgery.

At one time the French and German Schools had long and angry discussions regarding the comparative value of these two systems. Among the disciples of Duchenne, they had a preference for the Faradaic, and the Germans, under Remak, the galvanic, while in England and America, both are used, and are applicable each in its peculiar way. Now if muscular contraction is required it is only necessary to induce rapid interruption of the Faradaic current, and is best appreciated in a general electrization. So too the efficiency of the galvanic current is most remarkably seen when applied to the galvanic cautery. To produce the energetic caustic effect of the galvanic current, it is necessary to use such elements as may generate a quantity of electricity and combine them in such a way that the quantity produced shall be very large. With this current blood circulating in the living body as well as that which has been taken out, may be readily coagulated; another feature peculiar to this tissues, is, the acid passes to the positive, and the alkalies to the negative poles, and it has been proven, that the red corpuscles are dissolved at the negative poles. The remarkable tonic effect of both the galvanic and the faradic currents prove that it is essential to vitality, and when the system is depressed or impaired, can be supplied by electricity. Not only does it exist in the animal tissues, but also in the vegetable; and it has been ascertained by Dome, De Boise Raymond and others, that in apples and pears the current flows from the peduncle to the bud, and in fruit with large seed as the peaches and plums, from the bud to the peduncle. Says Dr. Wislizenus in a paper before the academy of medicine at St. Louis,—electricity has two daily tides, the high tide, between 9 and 12 A. M., and 6 and 9 P. M. The low tides between 2 and 5 P. M., and 1 and 5 A. M. The annual variations are as marked as the diurnal, and that it is most in excess in winter than summer. So in a storm you have a negative electricity and many persons will tell you that their corns or rheumatic pains are as susceptible as a barometer. In conclusion I will say that electricty has become one of the most powerful as well as useful agents we possess in the diagnosis and treatment of diseased humanity, and may become mischievous if injudiciously or improperly used.

